# Determination of APTT factor sensitivity – the misguiding guideline

**DOI:** 10.1111/ijlh.12109

**Published:** 2013-05-30

**Authors:** A S Lawrie, S Kitchen, M Efthymiou, I J Mackie, S J Machin

**Affiliations:** * Haemostasis Research Unit, Department of Haematology, University College LondonLondon, UK; † Department of Coagulation, Royal Hallamshire HospitalSheffield, UK

**Keywords:** APTT, plasma clotting factors

## Abstract

**Introduction:**

The Clinical and Laboratory Standards Institute (CLSI) has produced a guideline detailing how to determine the activated partial thromboplastin time’s (APTT) sensitivity to clotting factor deficiencies, by mixing normal and deficient plasmas. Using the guideline, we determined the factor sensitivity of two APTT reagents.

**Methods:**

APTTs were performed using Actin FS and Actin FSL on a Sysmex CS-5100 analyser. The quality of factor-deficient and reference plasmas from three commercial sources was assessed by assaying each of the clotting factors within the plasmas and by performing thrombin generation tests (TGT).

**Results:**

Testing samples from 50 normal healthy subjects gave a two-standard deviation range of 21.8–29.2 s for Actin FS and 23.5–29.3 s for Actin FSL. The upper limits of these ranges were subsequently used to determine APTT factor sensitivity. Assay of factor levels within the deficient plasmas demonstrated that they were specifically deficient in a single factor, with most other factors in the range 50–150 iu/dL (Technoclone factor VII-deficient plasma has 26 iu/dL factor IX). APTTs performed on mixtures of normal and deficient plasmas gave diverse sensitivity to factor deficiencies dependent on the sources of deficient plasma. TGT studies on the deficient plasmas revealed that the potential to generate thrombin was not solely associated with the levels of their component clotting factors.

**Conclusion:**

Determination of APTT factor sensitivity in accordance with the CLSI guideline can give inconsistent and misleading results.

## Introduction

The activated partial thromboplastin time (APTT) is a central component of any screen for haemostatic patency. The test is used to detect the deficiency of intrinsic clotting factors (VIII, IX, XI, or XII), the presence of lupus anticoagulant and the monitoring of unfractionated heparin therapy, although this latter function is becoming less common in many countries as unfractionated heparin has to a large extent been replaced with low molecular weight heparin. Consequently, the major role of the APTT at many centres is now detection of clotting factor deficiencies either in the form of a preoperative clotting screen or in patients who present with a history of a haemorrhagic diathesis. However, APTT reagents show different sensitivities to deficiencies of factors VIII, IX, XI and XII; this is thought to be because of differences in the activator or phospholipids used in the reagent 1,2.

Using samples from patients with congenital clotting factor deficiencies, we have previously assessed the sensitivity of two widely used APTT reagents, Actin FS and Actin FSL (Siemens Healthcare Diagnostics, Marburg, Germany) 3. Collection of clinical samples from patients with a single congenital coagulation factor deficiency (factors VIII, IX, XI or XII deficient) at appropriate levels of activity (i.e. 10–50%) taken into the sample tubes employed in a specific institute for a study of this type presents a logistical problem that would be beyond the capabilities of many general hospitals. Consequently, the Clinical and Laboratory Standards Institute (CLSI) Guideline 4 offers an alternative means of assessing APTT’s sensitivity to clotting factor deficiencies, which claims to circumvent this logistical problem.

The Guideline 4 requires that APTTs are performed on factor-deficient plasmas (i.e. factors VIII, IX, XI and XII) to which normal control plasma is added to give a range of relative potencies (<1 to 100%) for factors VIII, IX, XI and XII. However, as the APTT is a global test, the resultant clotting times would be influenced by the levels of all clotting factors within the spiked deficient plasma, not just the level of the clotting factor that had been adjusted. To investigate the influence of the factor-deficient plasma and normal plasma used, we determined the sensitivity of Actin FS and Actin FSL using three sources of plasma. The quality of factor-deficient plasma was also assessed in a thrombin generation test 5.

## Samples and Methods

Unless otherwise stated, all tests were performed on a Sysmex CS-5100 analyser (Sysmex UK Ltd, Milton Keynes, UK), and reagents were from Siemens Healthcare Diagnostics (Marburg, Germany). In addition to normal reference plasma and factor-deficient plasma from Siemens, lyophilized factor-deficient and reference plasmas were also obtained from Technoclone (Vienna, Austria). Frozen reference and factor-deficient plasmas (Cryocheck™) were obtained from Precision BioLogic (Nova Scotia, Canada).

Normal reference ranges for the two APTT reagents, Actin FS and Actin FSL, were confirmed using plasma samples from 50 healthy normal individuals. APTT quality control imprecision studies, using Actin FS and Actin FSL, were performed using commercial lyophilized normal (Control Plasma N [CPN]) and pathologically low plasmas (Control Plasma P [CPP] and CiTrol 2). Ten replicates of each preparation were tested on each of 5 days.

Factor assays were performed using either Actin FS (for factors VIII, IX, XI and XII) or Innovin (for factors II, V, VII and X). Fibrinogen concentration was measured using the Clauss technique. Levels of the component clotting factors within the Siemens, Technoclone and Cryocheck™ factor-deficient plasmas were determined relative to the Siemens reference preparation (Standard Human Plasma [SHP]). Similarly, the levels of clotting factors within the Technoclone and Cryocheck™ Reference plasmas were assayed relative to the SHP. The factor assays were all performed with samples tested at multiple dilutions, which facilitated an assessment of linearity and parallelism of dose response and thus detect possible inhibitory activity or falsely high results caused by sample activation 6.

The sensitivity of APTT reagents to coagulation factor deficiency was assessed using factor-deficient plasmas spiked with normal plasma to give samples with 90%, 80%, 70%, 60%, 50%, 40%, 30%, 20% and 10% activity of the single factor. For this process, Siemens deficient plasmas (factors VIII, IX, XI and XII) were spiked with Siemens SHP, Technoclone deficient plasmas with Technoclone Coagulation Reference Plasma and Cryocheck™ deficient plasmas with Cryocheck™ normal reference plasma. Each of the spiked plasma samples was assayed to confirm the activity of the single factor.

Thrombin generation test (TGT) studies were performed on the factors VIII-, IX-, XI- and XII-deficient plasma using the calibrated automated thrombogram (CAT) system as described by Hemker *et al*. 5 (Thrombinoscope BV, Maastricht, the Netherlands) in conjunction with the manufacturer’s PPP-low reagents, which gives a reaction concentrations of 1 pm tissue factor (TF)/4 μm phospholipid (PL) (Thrombinoscope BV). The thrombin generation test (TGT) provides information relating to the dynamics of thrombin generation (TG), and the TG curve is described in terms of the lag time, the time to peak, peak thrombin and the area under the TG curve, known also as the endogenous thrombin potential (ETP).

## Results

The normal reference ranges for APTTs performed using Actin FS and Actin FSL were confirmed by testing the plasma from 50 healthy normal individuals. This process gave a mean ± two-standard deviation range of 21.8–29.2 s for Actin FS and 23.5–29.3 s for Actin FSL. The upper limits of these ranges were subsequently used to determine APTT factor sensitivity. Reproducibility of the APTT assessed using commercial lyophilized normal and pathologically low plasmas yielded similarly low levels of imprecision for both Actin FS and Actin FSL (Table 1).

**Table 1:** APTT imprecision using three levels of commercial quality control plasma

	Control Plasma N			Ci-Trol Level 2			Control Plasma P		
Reagents	Mean (s)	SD (s)	CV (%)	Mean (s)	SD (s)	CV (%)	Mean (s)	SD (s)	CV (%)
Actin FS	26.1	0.12	0.48	44.8	0.23	0.50	51.3	0.68	1.33
Actin FSL	26.2	0.09	0.36	42.3	0.22	0.51	74.7	1.51	2.03

Factor assays performed to assess the levels of the component clotting factors within the Siemens, Technoclone and Cryocheck™ factor-deficient plasmas confirmed normal activity (50–150 iu/dL) of each of the clotting factors present (Table 2). Only one of the deficient plasmas was found to have an equivocal level of a relevant clotting factor (Technoclone factor VIII-deficient plasma, factor XI level 50 iu/dL) (Table 2).

**Table 2:** Levels of coagulation factors in commercial deficient plasmas

Def Plasma	Fg (g/L)	FII (iu/dL)	FV (iu/dL)	FVII (iu/dL)	FVIII (iu/dL)	FIX (iu/dL)	FX (iu/dL)	FXI (iu/dL)	FXII (iu/dL)
Technoclone									
FII	2.75	<1.0	92	125	79	105	104	77	102
FV	3.31	109	<1.0	106	72	124	116	99	115
FVII	2.88	109	91	<1.0	83	26	103	83	102
FVIII	2.18	96	89	126	<1.0	98	105	50	63
FIX	3.14	85	123	72	100	<1.0	107	67	98
FX	2.44	102	80	109	106	84	<1.0	61	113
FXI	2.34	115	111	138	82	92	91	<1.0	73
FXII	2.31	95	91	122	92	112	96	82	<1.0
Ref Plasma	2.75	98	93	114	98	111	99	87	93
Siemens									
FII	2.15	<1.0	92	97	74	79	89	74	93
FV	1.90	82	<1.0	85	70	76	84	76	85
FVII	2.13	82	86	<1.0	78	78	80	76	83
FVIII	1.95	78	81	85	<1.0	74	80	67	55
FIX	2.34	90	103	102	68	<1.0	98	83	86
FX	2.44	94	95	103	74	92	<1.0	84	95
FXI	1.99	84	91	86	73	77	88	<1.0	84
FXII	2.37	97	97	105	80	93	99	82	<1.0
Ref Plasma	2.48	94	87	94	90	93	92	88	98
CryoCheck									
FII	2.18	<1.0	92	117	95	116	114	94	110
FV	2.84	110	<1.0	123	110	123	120	102	113
FVII	2.84	109	103	<1.0	105	118	114	102	116
FVIII	2.13	117	106	137	<1.0	130	126	111	107
FIX	2.84	111	106	131	105	<1.0	127	97	116
FX	2.75	108	88	124	96	110	<1.0	90	112
FXI	2.93	109	114	115	106	115	122	<1.0	107
FXII	2.75	112	79	144	103	123	123	99	<1.0
Ref Plasma	2.63	112	120	123	109	123	123	104	107

The highlighted coagulation factor levels are either equivocal or below the normal reference range.

**Figure 1:**
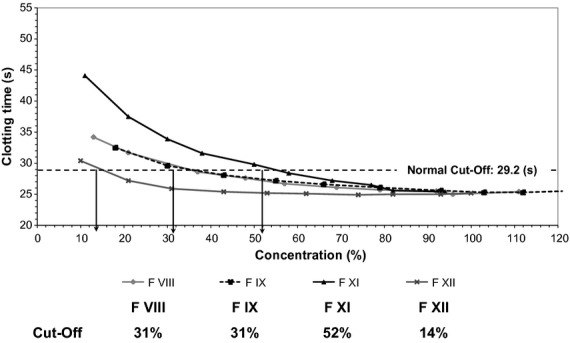
Determination of Actin FS Factor Sensitivity using CryoCheck Frozen Factor-Deficient Plasma.

**Figure 2:**
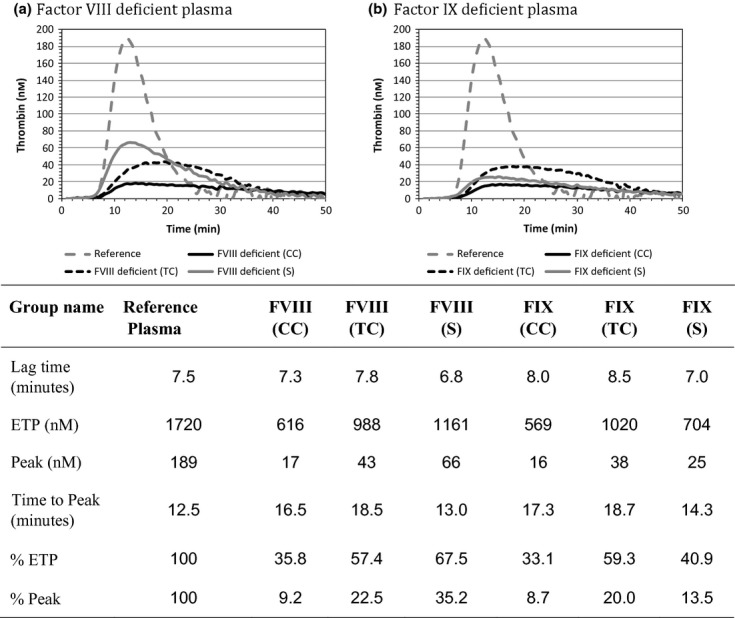
Thrombin generation curves for FVIII- and FIX-deficient plasmas – 1 pm TF. CC: Cryocheck, TC: Technoclone, S: Siemens. ETP: Endogenous thrombin potential. % ETP and % Peak were calculated by expressing the specific activity of these parameters as percentage of the normal reference plasma processed in the same assay.

Using the upper limit of the APTT reference ranges, the sensitivity of APTT reagents to a single coagulation factor deficiency was determined with factor-deficient plasma spiked with normal plasma to give samples of 90%, 80%, 70%, 60%, 50%, 40%, 30%, 20% and 10% activity (an example of which is seen in Figure 1). Data from this study demonstrated that APTT’s sensitivity to clotting factor deficiency was influenced by the source of deficient and normal reference plasma used (Table 3). Thrombin generation studies revealed that the potential to generate thrombin was not solely related to the levels of clotting factors present in the deficient plasma (Figure 2).

**Table 3:** Level of intrinsic clotting factor detected at Upper Limit of APTT

Deficient Plasma	APTT Upper Limit (s)	Actin FS 29.2	Actin FSL 29.3
Technoclone	F. VIII Activity (%)	68	66
Siemens		73	62
CryoCheck		32	32
Technoclone	F. IX Activity (%)	58	46
Siemens		76	56
CryoCheck		32	25
Technoclone	F. XI Activity (%)	58	46
Siemens		71	50
CryoCheck		52	25
Technoclone	F. XII Activity (%)	22	22
Siemens		78	50
CryoCheck		14	18

## Discussion

Central to the role of the APTT is an understanding of its ability to detect clotting factor deficiencies. As the APTT is a global clotting test, a normal APTT does not necessarily indicate that all clotting factors within the intrinsic coagulation pathway (factors VIII, IX, XI and XII) are normal. A deficiency of one intrinsic clotting factor can be masked by an elevated level of another. For example, FVIII elevated by an acute phase response can mask a mild or even moderate deficiency of factor IX or XI. For this reason, when defining the sensitivity of an APTT reagent to a single-factor deficiency, it is essential to determine the quality of the samples used for this process by measuring all intrinsic clotting factors in the samples and the precision with which the instrumentation measures the APTT.

Our imprecision data (Table 1) demonstrated highly reproducible APTTs at the three levels of quality control with both the Actin FS and Actin FSL reagents. Importantly, we also demonstrated that each of the factor-deficient plasmas used in the study was specifically deficient in a single clotting factor while the other component clotting factors were present at normal levels (50–150 iu/dL), as shown in Table 2. Consequently, when the deficient plasmas were spiked with normal plasmas to yield a range of relative potencies, as suggested by the CSLI guideline 4, it was interesting to observe the wide range of clotting factor activities that were associated with the APTT upper limit of the normal reference range using Actin FS and Actin FSL (Table 3).

As would be anticipated, when TGT studies were performed using a TF trigger, the FXII-deficient plasmas gave a normal thrombin generation (TG) curve while the FX-, FVII-, FV- and FII-deficient plasmas gave no TG under the test conditions described (data not shown). However, the FVIII- and FIX-deficient plasma TGT results were intriguing because those plasmas with the highest levels of component clotting factors (i.e. Cryocheck™, frozen plasma) gave the lowest level of TG while the plasma with the lowest levels of clotting factors (i.e. Siemens, lyophilized plasma) gave the highest level of TG. This suggested that the potential to generate thrombin was not solely associated with the level of component clotting factors; it could be speculated that lyophilized deficient plasmas may contain a procoagulant material 7 that only exerts an effect in TGT or in a test system such as that described in the CSLI guideline 4. This does not mean lyophilized plasmas are unsuitable for their intended use (i.e. one-stage clotting assays), as each of the deficient plasmas produced sensitive dose–response curves in one-stage clotting assays.

We conclude that determination of APTT factor sensitivity performed in accordance with CLSI Document H47-A2 4 can give inconsistent and misleading results, and this approach should not be used in routine laboratory practice. Consequently, detection limits should either be determined by the reagent manufacturers for specific instrument/reagent systems or by individual laboratories using well-characterized samples from patients with inherited coagulation deficiencies. We would suggest testing a minimum of 20 deficient samples with potencies evenly distributed in the range 10–50% for each of the intrinsic coagulation factors (factors VIII, IX, XI and XII).
